# Microwave synthesis of high-quality and uniform 4 nm ZnFe_2_O_4_ nanocrystals for application in energy storage and nanomagnetics

**DOI:** 10.3762/bjnano.7.126

**Published:** 2016-09-27

**Authors:** Christian Suchomski, Ben Breitung, Ralf Witte, Michael Knapp, Sondes Bauer, Tilo Baumbach, Christian Reitz, Torsten Brezesinski

**Affiliations:** 1 Institute of Physical Chemistry, Justus-Liebig-University Giessen, Heinrich-Buff-Ring 17, 35392 Giessen, Germany; 2 Battery and Electrochemistry Laboratory, Institute of Nanotechnology, Karlsruhe Institute of Technology, Hermann-von-Helmholtz-Platz 1, 76344 Eggenstein-Leopoldshafen, Germany; 3 Institute of Nanotechnology, Karlsruhe Institute of Technology, Hermann-von-Helmholtz-Platz 1, 76344 Eggenstein-Leopoldshafen, Germany; 4 Institute for Applied Materials, Karlsruhe Institute of Technology, Hermann-von-Helmholtz Platz 1, 76344 Eggenstein-Leopoldshafen, Germany; 5 Institute for Photon Science and Synchrotron Radiation, Karlsruhe Institute of Technology, Hermann-von-Helmholtz-Platz 1, 76344 Eggenstein-Leopoldshafen, Germany

**Keywords:** 1-phenylethanol route, lithium-ion battery, nanomagnetism, nanoparticles, nonaqueous sol–gel synthesis, zinc ferrite

## Abstract

Magnetic nanocrystals with a narrow size distribution hold promise for many applications in different areas ranging from biomedicine to electronics and energy storage. Herein, the microwave-assisted sol–gel synthesis and thorough characterization of size-monodisperse zinc ferrite nanoparticles of spherical shape is reported. X-ray diffraction, ^57^Fe Mössbauer spectroscopy and X-ray photoelectron spectroscopy all show that the material is both chemically and phase-pure and adopts a partially inverted spinel structure with Fe^3+^ ions residing on tetrahedral and octahedral sites according to (Zn_0.32_Fe_0.68_)^tet^[Zn_0.68_Fe_1.32_]^oct^O_4±δ_. Electron microscopy and direct-current magnetometry confirm the size uniformity of the nanocrystals, while frequency-dependent alternating-current magnetic susceptibility measurements indicate the presence of a superspin glass state with a freezing temperature of about 22 K. Furthermore, as demonstrated by galvanostatic charge–discharge tests and ex situ X-ray absorption near edge structure spectroscopy, the as-prepared zinc ferrite nanocrystals can be used as a high-capacity anode material for Li-ion batteries, showing little capacity fade – after activation – over hundreds of cycles. Overall, in addition to the good material characteristics, it is remarkable that the microwave-based synthetic route is simple, easily reproducible and scalable.

## Introduction

Spinel ferrites of the general formula MFe_2_O_4_ constitute a well-known class of materials with unique physical and chemical properties and they hold promise for use in various fields of nanotechnology [1–2]. One of those properties is magnetism. In recent years, it has been shown that particularly size-monodisperse nanoparticles provide an attractive platform for future magnetic data storage and theranostics (that is, imaging and therapy in biomedicine). The challenges and prospects in using Fe-based nanoparticles for such applications have been described in excellent papers elsewhere and will therefore not be discussed here [3–8]. The magnetic properties of spinel ferrites are known to be strongly dependent upon their size [9–11]. Part of the reason for this is that, for example, significant spin disorder occurs when the particle size is reduced to the nanometer level. Nevertheless, the cation site occupancy also plays a central role in the magnetism (exchange interactions) and usually exhibits a variety among different synthesis methods. Overall, this means that the magnetic properties can be tailored to some extent by means of the preparation conditions.

Furthermore, spinel ferrites have been shown to be capable of reacting electrochemically with Li to form Li_2_O and reduced metal phases [12–14]. However, bulk forms of these materials have not proven to be of interest for battery applications because of sluggish conversion reaction kinetics and fast capacity decay on cycling. Since small-size particles can better accommodate the strain from the Li insertion, nanocrystallinity seems to be playing the key role to achieving “good” charge storage characteristics or, in other words, high performance.

Various synthetic methods to produce single-phase spinel ferrite nanoparticles have been reported in the literature, including hydrothermal, mechanochemical and sol–gel routes (to name but a few) [15–19], and in particular solution-phase approaches seem promising with respect to exercising control over size and shape [20–21]. As an example, the preparation of uniform 4–8 nm diameter MFe_2_O_4_ (M = Fe, Co, Mn, Ni) nanoparticles has been achieved by microwave-assisted nonaqueous sol–gel synthesis using benzyl alcohol as a high-boiling solvent [22–23]. Inspired by this work, we show here that high-quality and size-monodisperse zinc ferrite (referred to as ZFO in the following) nanocrystals can be produced via facile microwave synthesis by the use of *rac*-1-phenylethanol. 1-Phenylethanol exhibits excellent solvent properties – especially for anhydrous zinc acetate – and therefore ensures that both salt precursors are completely dissolved, so that the formation of impurity phases, such as Fe_3_O_4_, can be avoided.

## Experimental

### Synthesis

In a typical synthesis, anhydrous zinc acetate (91.7 mg, 99.99%, Sigma-Aldrich) was dissolved by sonication in dry *rac*-1-phenylethanol (15 mL, 98%, Sigma-Aldrich). Then, iron(III) acetylacetonate (353.2 mg, 99.9%, Sigma-Aldrich) was added, followed by sonication for 5 min. The resulting dark red solution was transferred to a borosilicate vial (30 mL), sealed with a screw cap and heated at 200 °C under microwave irradiation for 25 min. The stirring rate was set to 300 rpm. After quenching with compressed cold air, the ZFO nanoparticles were precipitated by addition of n-pentane and collected by centrifugation, followed by washing twice with a solution of acetone and ethanol. Finally, the obtained brown powder was allowed to dry at room temperature.

Microwave syntheses were performed using both Monowave 300 and Masterwave BTR reactors (*f* = 2.45 GHz, Anton Paar Germany GmbH) equipped with either one or two 850 W magnetrons. The temperature was monitored with a ruby thermometer (fiber-optic probe) placed inside the reaction vial (Monowave 300) and with an IR sensor (Pt100) mounted at the bottom of the Masterwave BTR cavity. Pressure sensing was accomplished by a hydraulic sensor.

### Electrode processing

ZFO nanoparticle electrodes were prepared by casting a water slurry containing 79 wt % ZFO, 11 wt % Super C65 carbon black additive (Timcal) and 10 wt % Selvol 425 poly(vinyl alcohol) (Sekisui) onto Cu foil (Gould Electronics), followed by drying in vacuum at 80 °C for 12 h. The areal loading was 2.4 mg_ZFO_/cm^2^ on average. Coin-type cells with 600 µm-thick Li metal foil (Rockwood Lithium Inc.) and glass microfiber film separator (Whatman, GF/D grade) were assembled inside an argon-filled glovebox (MBraun) with [O_2_] and [H_2_O] < 1 ppm. The electrolyte was 1 M LiPF_6_ in fluoroethylene carbonate and ethyl methyl carbonate (1:1 weight ratio). The cycling performance was evaluated at rates ranging from C/10 to C/2 (1C = 1000.5 mA/g_ZFO_).

### Characterization

Powder X-ray diffraction patterns were recorded on a STOE diffractometer with a Mo Kα_1_ radiation source, focusing Ge 111 monochromator and a Dectris Mythen strip detector. Rietveld refinement was performed by use of the FullProf software. X-ray photoelectron spectroscopy data were obtained on a VersaProbe PHI 5000 Scanning ESCA Microprobe from Physical Electronics with an Al Kα radiation source and a hemispherical electron energy analyzer. The C 1s signal from adventitious hydrocarbon at 284.8 eV was used as the energy reference to correct for charging. Mössbauer spectroscopy was performed in transmission geometry using a constant acceleration spectrometer with a ^57^Co radiation source embedded in a Rh matrix. The center shifts are quoted relative to α-Fe foil at room temperature. The spectra were analyzed using the WinNORMOS software [24]. Transmission electron microscopy was performed on a Tecnai G2-F20ST microscope (FEI) operated at 200 keV. The bright-field images were analyzed using the iTEM software. Thermogravimetric analysis was performed on a Netzsch STA 409 PC. The thermobalance was coupled to a Balzers QMG 421 quadrupole mass spectrometer. The ionization energy was 70 eV. Gas chromatography-mass spectrometry was performed on an Agilent 6890 gas chromatograph equipped with an Agilent 5973 MSD. Diffuse reflectance ultraviolet–visible spectra were collected on a Lambda 750 UV–vis–NIR spectrophotometer (PerkinElmer) equipped with a Praying Mantis diffuse reflectance accessory. An MPMS XL-5 superconducting quantum interference device magnetometer (Quantum Design) was used for magnetic susceptibility measurements in the field range from +45 kOe (+4.5 T) to −45 kOe (−4.5 T). Electrochemical measurements were performed in a BINDER cooled incubator using a MACCOR Series 4000 cycler (Tulsa). 2D imaging of chemical phase transformations at the nanoscale by full-field transmission X-ray microscopy using a Carl Zeiss TXM and the corresponding data treatment steps are described elsewhere [25]. To determine the oxidation state of Fe, several different Fe-based compounds were measured and used as the references. The fitting of X-ray absorption near edge structure spectra is based on a least-squares linear combination of reference spectra and was carried out by use of TXM-XANES Wizard after normalization [26]. The quality of fits was assessed by the misfit factor, *R*.

## Results and Discussion

Highly crystalline ZFO nanoparticles were prepared by microwave-assisted nonaqueous sol–gel synthesis using anhydrous zinc acetate and iron(III) acetylacetonate as the precursors and *rac*-1-phenylethanol as a solvent. Details on the formation mechanism from gas chromatography-mass spectrometry (GC-MS) are given in Supporting Information File 1, Figure S1–S4 and Table S1.

**Figure 1:**
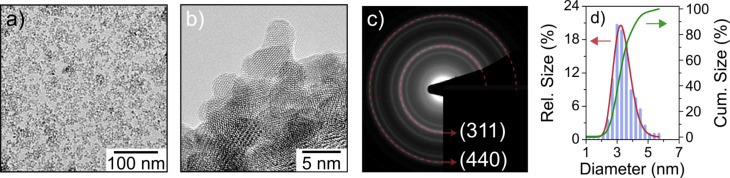
Electron microscopy of as-prepared ZFO nanoparticles. (a) Bright-field TEM image. (b) HRTEM image and (c) SAED pattern demonstrating the crystallinity. Note that only the most intense diffraction rings are indexed in (c). (d) Relative and cumulative particle size distributions. The red line is a log-normal fit to the data.

The size and shape of the ZFO nanoparticles was investigated by means of transmission electron microscopy (TEM). The low-magnification bright-field TEM image in Figure 1a shows that they are spherical in shape with a narrow size distribution around 4 nm. Both high-resolution TEM (HRTEM, Figure 1b) and selected-area electron diffraction (SAED, Figure 1c) demonstrate the high crystallinity of the ZFO nanoparticles. In addition, SAED indicates that the sol–gel derived material is single-phase and adopts a cubic structure. Figure 1d presents the size distribution obtained by particle counting from TEM images. These data can be fitted by a log-normal distribution with a mean of 3.3 nm and standard deviation of 0.2 nm.

**Figure 2:**
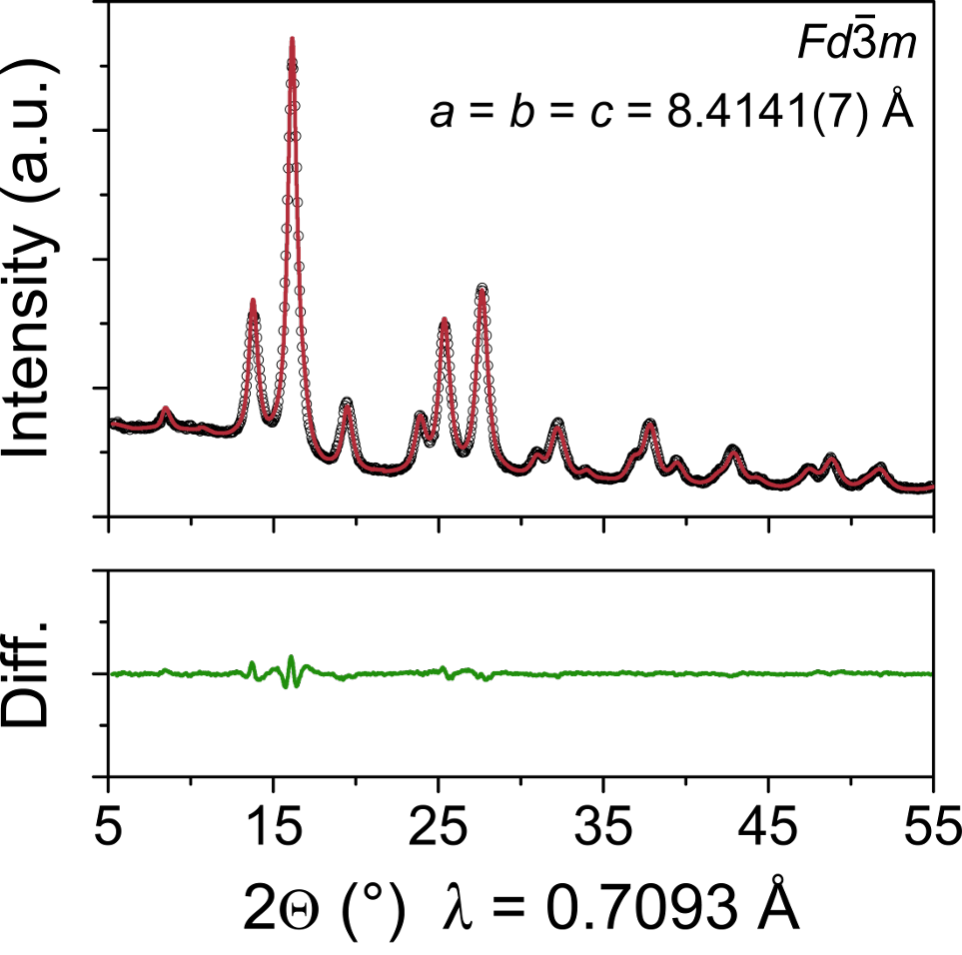
Observed (open circles) and calculated (red line) XRD patterns of as-prepared ZFO nanoparticles. The difference profile of the fit is shown in green.

The microstructure of the as-prepared ZFO nanoparticles was analyzed in more detail by powder X-ray diffraction (XRD). The XRD pattern in Figure 2 corroborates the SAED results, showing only reflections characteristic of spinel-type franklinite with 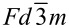 space group (ICDD-JCPDS card no. 22-1012) [27]. A fit applying a modified Thompson–Cox–Hastings pseudo-Voigt profile function revealed lattice parameters of *a* = *b* = *c* = 8.4141(7) Å and a crystallite size of 4(1) nm – in line with the size distribution from particle counting. The quality of the refinement (NIST Si 640c was used as the instrument line-broadening standard) was assessed by the magnitude of the weighted profile *R*-factor (*R*_wp_ = 8.0%) and the goodness-of-fit parameter (χ^2^ = 0.463). The fact that the observed and calculated XRD patterns match with each other well and the latter values are low implies that the fit can be considered good.

As mentioned in the introduction, ZFO belongs to the spinel ferrite family of the general formula AB_2_O_4_. The inversion parameter, λ, typically serves as a measure of the cation distribution according to (A_1−λ_B_λ_)^tet^[A_λ_B_2−λ_]^oct^O_4_. Bulk ZFO has been shown to exhibit virtually no inversion (λ ≈ 0) and thus is considered a normal spinel [28–29]. In this structure, the A cations occupy the tetrahedral 8a sites (Wyckoff notation), while the B cations reside on two equivalent octahedral 16d sites. In contrast, the octahedral coordination sites are randomly occupied in a 1:1 ratio by the A and B cations and the tetrahedral sites are only occupied by the B cations in inverse spinels (λ = 1). In both cases, the O^2−^ ions form a cubic close-packed structure and reside on 32e sites. However, nanoscale spinel ferrites prepared by wet chemical methods are known to often have a partially inverted structure [30–32]. Therefore, the cation site occupancy in the ZFO nanoparticles employed in this work was expected to differ from that of bulk material.

**Figure 3:**
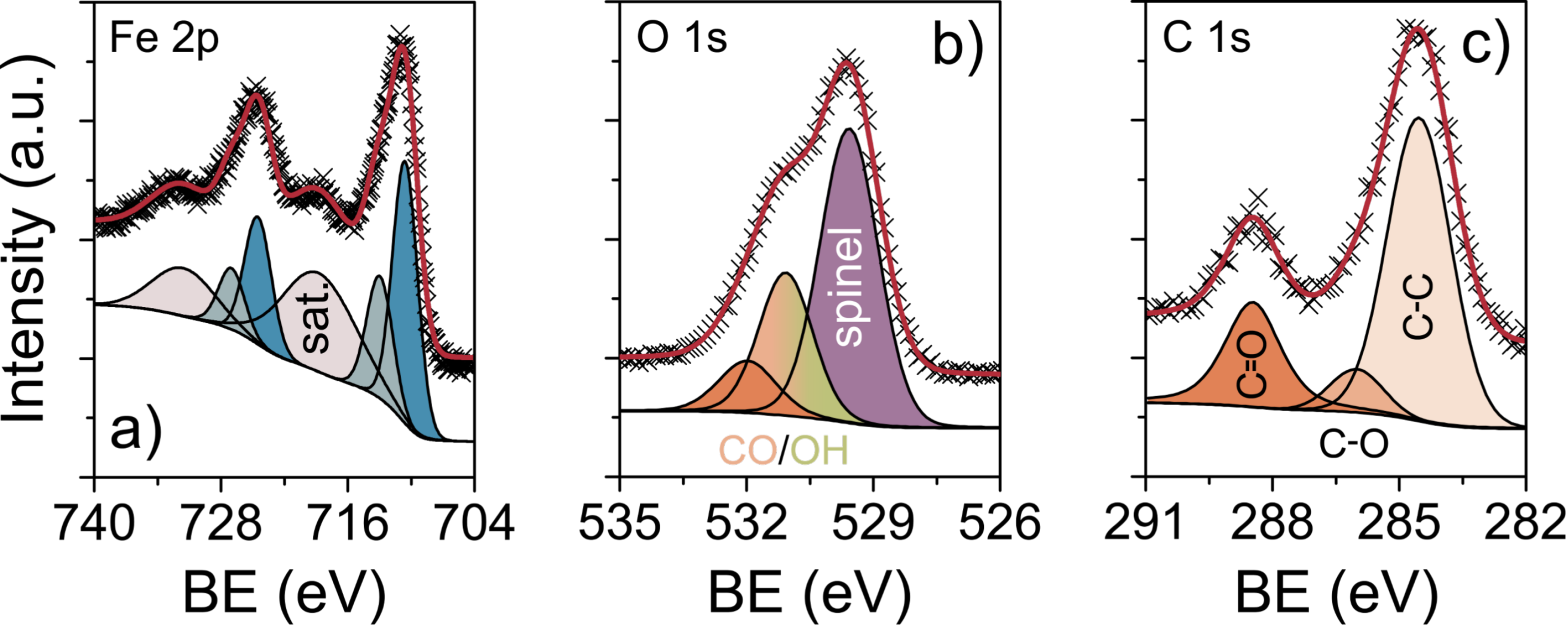
XPS spectra of the (a) Fe 2p, (b) O 1s and (c) C 1s core levels of as-prepared ZFO nanoparticles. The peaks in gray, blue, violet and orange/green correspond to tetragonal Fe^3+^, octahedral Fe^3+^, lattice oxygen as well as hydroxyl oxygen and different organic compounds containing carbon–oxygen functionalities. The red lines are the sum of the peak fits.

The distribution of Fe among the tetrahedral and octahedral sites was studied by X-ray photoelectron spectroscopy (XPS) and Mössbauer spectroscopy. Figure 3a–c presents detailed XPS spectra of the Fe 2p, O 1s and C 1s core level regions. The Fe 2p spectrum shows a single doublet with strong satellite peaks around 8 eV higher in binding energy than the main peaks. This result is characteristic of Fe in the Fe(III) state [33–34]. The apparent asymmetry of the Fe 2p peaks suggests that the inversion parameter must be greater than zero. The main peaks at binding energies of (724.59 ± 0.05) eV and (710.65 ± 0.05) eV for the 2p_1/2_ and 2p_3/2_ orbital lines, respectively, correspond to octahedral Fe^3+^ ions and the minor peaks at (727.12 ± 0.05) eV and (713.05 ± 0.05) eV to tetrahedral Fe^3+^ ions [35–37]. The inversion parameter was determined to be 0.68 by comparing the areas under the peaks. Accordingly, the formula can be expressed as (Zn_0.32_Fe_0.68_)^tet^[Zn_0.68_Fe_1.32_]^oct^O_4±δ_. The deconvolution of the O 1s spectrum identified three different oxygen bonding states. The main peak at (529.60 ± 0.05) eV corresponds to lattice oxygen and the minor peaks at higher binding energies of (531.11 ± 0.05) eV and (532.07 ± 0.05) eV can be assigned to hydroxyl oxygen/oxygen from C–O and C=O functionalities, respectively, with the latter originating from surface ligands. The C 1s spectrum can also be fitted into three peaks at (284.56 ± 0.05) eV, (286.06 ± 0.05) eV and (288.49 ± 0.05) eV. We ascribe the main peak centered at 284.6 eV to sp^3^-hybridized carbon (C–C); the minor peaks at higher binding energies arise from organic compounds containing C–O and C=O bonds, respectively. The fact that the as-prepared nanoparticles are not “naked” was also confirmed by thermogravimetric analysis-mass spectrometry (TGA-MS). The TGA-MS data of vacuum-dried material in Supporting Information File 1, Figure S5 indicate a mass loss of 13% in the temperature range between 150 °C and 400 °C due to release of water and combustion of acetate and acetylacetonate ligands.

**Figure 4:**
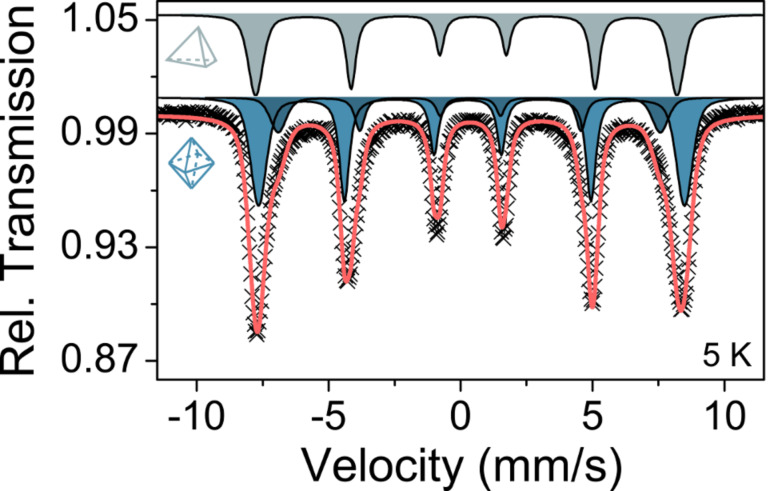
Low-temperature Mössbauer data of as-prepared ZFO nanoparticles. The gray spectrum represents Fe^3+^ on tetrahedral sites, while the blue spectra correspond to Fe^3+^ residing on octahedral sites. The red line is the sum of the different sub-spectra.

To verify the XPS results, ^57^Fe Mössbauer spectroscopy was performed on the ZFO nanoparticles. A representative spectrum measured at 5 K is provided in Figure 4. It reveals a sextet pattern because of the presence of magnetic ordering. This is in accordance with both the magnetometry data below and temperature-dependent Mössbauer spectra shown in Supporting Information File 1, Figure S6. The low-temperature data can be fitted reasonably well with three sub-spectra, in line with findings by Chinnasamy et al. on nanoscale ZFO prepared by ball-milling [10]. They identified two octahedral B-site components (oct-1, oct-2) due to different local environments of the Fe^3+^ ions and one tetrahedral A-site component (tet) using Mössbauer spectroscopy with and without an external magnetic field. Table 1 summarizes the fitted parameters, which agree with the ratio of tetrahedral to octahedral Fe in the partially inverted spinel structure from XPS. In addition, Figure 4 confirms that all Fe ions are in the Fe(III) state. This is also corroborated by the fact that the formation of acetophenone can be ruled out on the basis of the GC-MS data (Supporting Information File 1, Figure S1). The latter compound is found in the microwave synthesis of Fe_3_O_4_ nanoparticles under identical conditions due to partial oxidation of 1-phenylethanol (data not shown), which is accompanied by the reduction of Fe^3+^ to Fe^2+^.

**Table 1:** Summary of fitted Mössbauer parameters.^a^

Site	CS (mm/s)	QS (mm/s)	*B*_Hf_ (T)	Area ratio

oct-1	0.45(1)	0.16(1)	50.0(5)	0.45(2)
oct-2	0.46(1)	−0.04(1)	45.0(1)	0.23(1)
tet	0.45(1)	−0.26(1)	49.6(1)	0.32(2)

^a^CS: center shift relative to α-Fe at 298 K, QS: quadrupole splitting, *B*_Hf_: magnetic hyperfine splitting.

**Figure 5:**
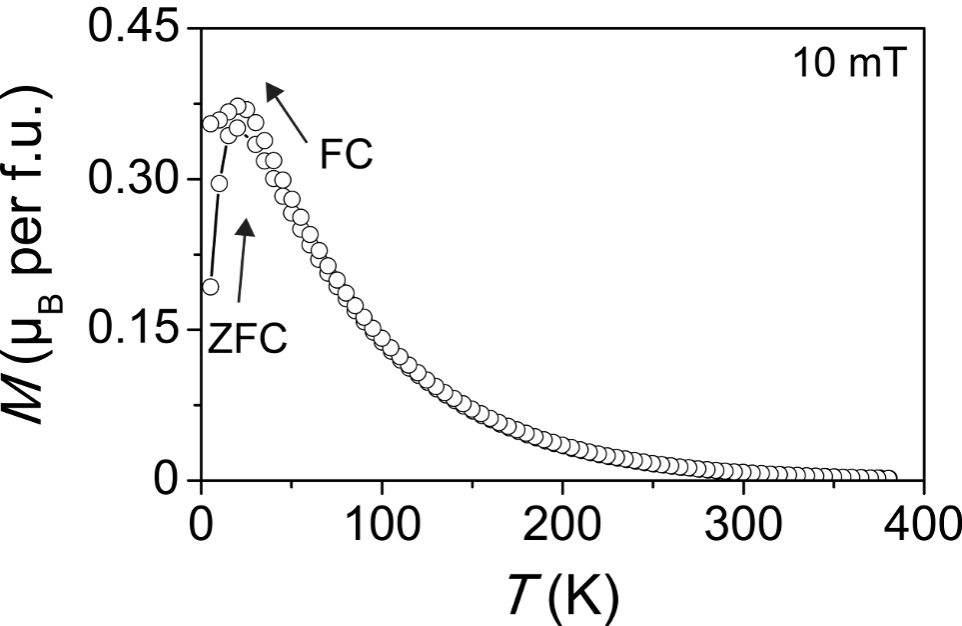
Direct-current SQUID magnetometry of as-prepared ZFO nanoparticles. ZFC/FC curves measured with µ_0_*H*_DC_ = 10 mT.

The magnetic properties were thoroughly investigated by both direct-current (DC) and alternating-current (AC) superconducting quantum interference device (SQUID) magnetometry. Zero-field-cooled (ZFC) and field-cooled (FC) curves obtained on the as-prepared ZFO nanoparticles at an applied field of 10 mT are shown in Figure 5. As seen, the magnetic moment continuously increases until a maximum, *T*_max_, is reached at about 22 K. The fact that this maximum is rather sharp supports the size uniformity of the particles with a similar magnetic anisotropy. Upon further cooling, the FC curve diverges from the ZFC curve and the material exhibits ferrimagnetic behavior.

**Figure 6:**
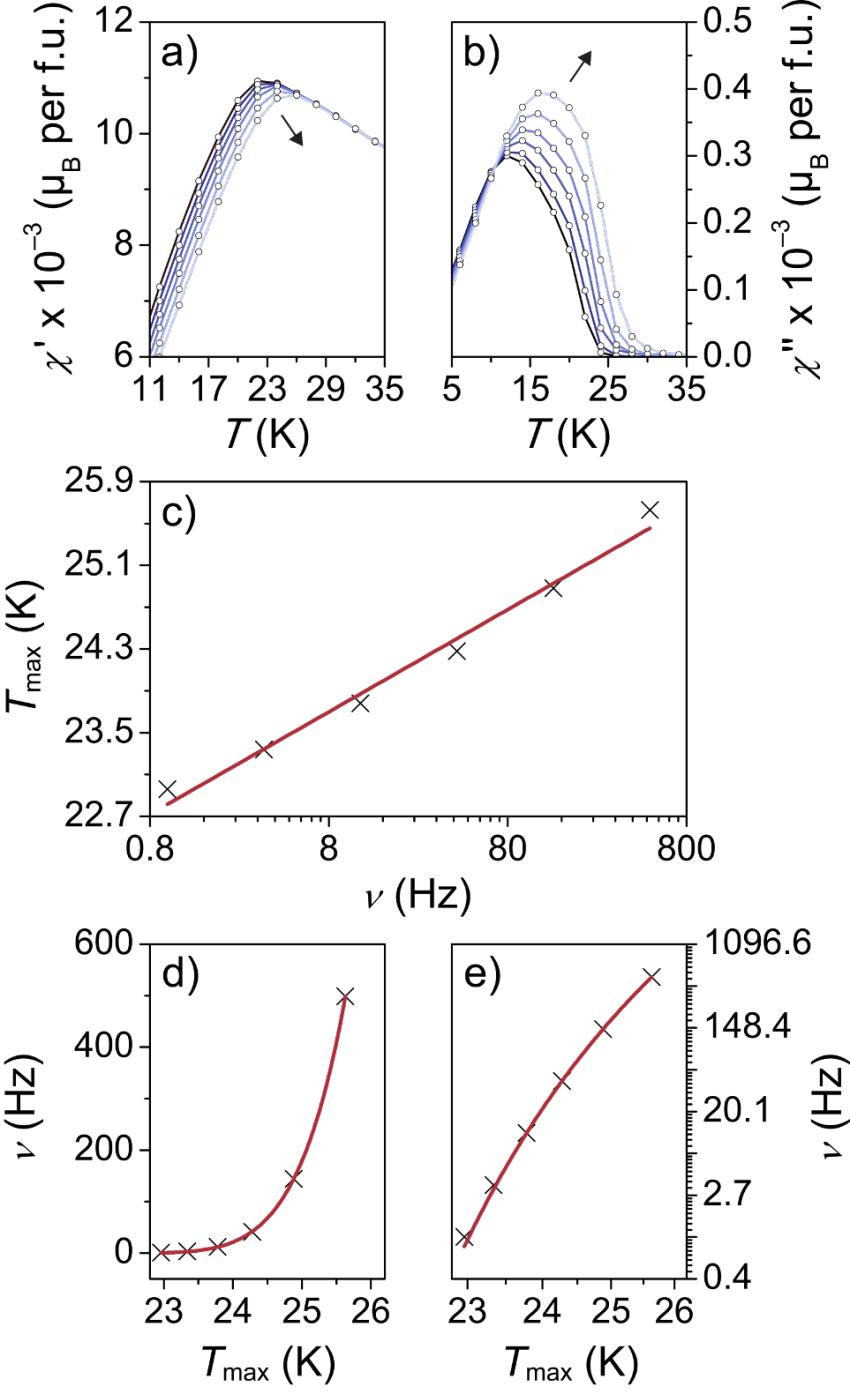
Alternating-current SQUID magnetometry of as-prepared ZFO nanoparticles with µ_0_*H*_AC_ = 0.35 mT. (a) In-phase and (b) out-of-phase parts of the magnetic susceptibility. (c–e) Frequency dependence of the peak temperature in the χ’(*T*) curve. The red lines are fits to the data according to *p* = Δ*T*_max_/[*T*_max_ × Δlog(ν)] in (c), ν = ν_0_ × [(*T*_max_ – *T*_0_)/*T*_0_]*^z^*^υ^ in (d) and ν = ν_0_ × exp[*–E*_a_/*k*_B_(*T*_max_ – *T*_0_)] in (e).

To determine whether *T*_max_ can be associated with either a freezing temperature, *T*_f_, for spin glasses or a blocking temperature, *T*_B_, for superparamagnetic particles, frequency-dependent AC magnetic susceptibility measurements were carried out. We note that single-domain particle ensembles, in which interparticle interactions are non-negligible, are referred to as superspin glasses in the following [38–39]. The amplitude of the AC field was set to µ_0_*H*_AC_ = 0.35 mT and the driving frequency, ν, was varied from 1 Hz to 500 Hz. Figure 6a,b shows the in-phase, χ’, and out-of-phase, χ’’, parts of the complex susceptibility (with χ_AC_ = χ’ – iχ’’) in units of µ_B_ per formula unit (f.u.). In the plot of χ’ vs *T*, *T*_max_ shifts to higher temperatures but lower magnetization values with increasing frequency. In contrast, the χ’’(*T*) curve shows an increase in susceptibility with increasing frequency. Unfortunately, such frequency and temperature dependencies are usually found for both (super)spin glasses and superparamagnets. Therefore, the relative variation of *T*_max_ (defined as the peak temperature in the χ’(*T*) curve) per frequency decade was analyzed in more detail. First, the data were fitted according to *p* = Δ*T*_max_/[*T*_max_ × Δlog(ν)], where *p* is the frequency sensitivity. From the fit (Figure 6c), we obtained *p* = 0.044, which is in the range observed for canonical spin glasses [35,39–40]. The frequency dependence of the peak temperature can also be described by a critical power law according to ν = ν_0_ × [(*T*_max_ – *T*_0_)/*T*_0_]*^z^*^υ^ with τ_0_ = 1/2πν_0_ and *T*_r_ = (*T*_max_ *– T*_0_)/*T*_0_, where τ_0_ is the microscopic spin relaxation time, *T*_r_ is the reduced temperature and *z*υ is the dynamical exponent [39,41]. The best fit (Figure 6d) was obtained with τ_0_ = 1.43 × 10^–8^ s, *T*_0_ = 21.7 K and *z*υ = 5.9. Both the value of τ_0_ and *z*υ falls within the range expected for canonical spin glasses [42]. Lastly, the data were fitted according to the Néel–Brown equation (ν = ν_0_ × exp[*KV*/*k*_B_*T*_max_)] for ideal non-interacting superparamagnetic particle ensembles, where *K* is the effective uniaxial magnetic anisotropy, *V* is the particle volume and *k*_B_ is the Boltzmann’s constant [43]. As shown in Supporting Information File 1, Figure S7, the frequency dependence of the peak temperature does not follow Néel–Brown model, which is supported by the finding that the Néel–Arrhenius relation gave an unphysically large value of *E*_a_/*k*_B_ (1365 K). This has also been observed for other materials with (super)spin glass behavior and thus confirms the conclusion of spin glass freezing rather than superparamagnetic blocking [44–45]. For slightly interacting nanoparticle ensembles, the frequency dependence should follow the empirical Vogel–Fulcher law (ν = ν_0_ × exp[*–E*_a_/*k*_B_(*T*_max_ – *T*_0_)] with –*E*_a_ = *KV*), where *T*_0_ is the interparticle interaction strength parameter and *E*_a_ is the activation energy [46–47]. From the best fit to the data (Figure 6e), we obtained τ_0_ = 1.21 × 10^–8^ s, *T*_0_ = 18.8 K and *E*_a_/*k*_B_ = 70.2 K. These values are in good agreement with those reported in the literature and those obtained from the power law plot in Figure 6d [38,48]. Overall, the DC and AC magnetization data reveal the signatures of a superspin glass state with a freezing temperature *T*_f_ ≈ 22 K.

**Figure 7:**
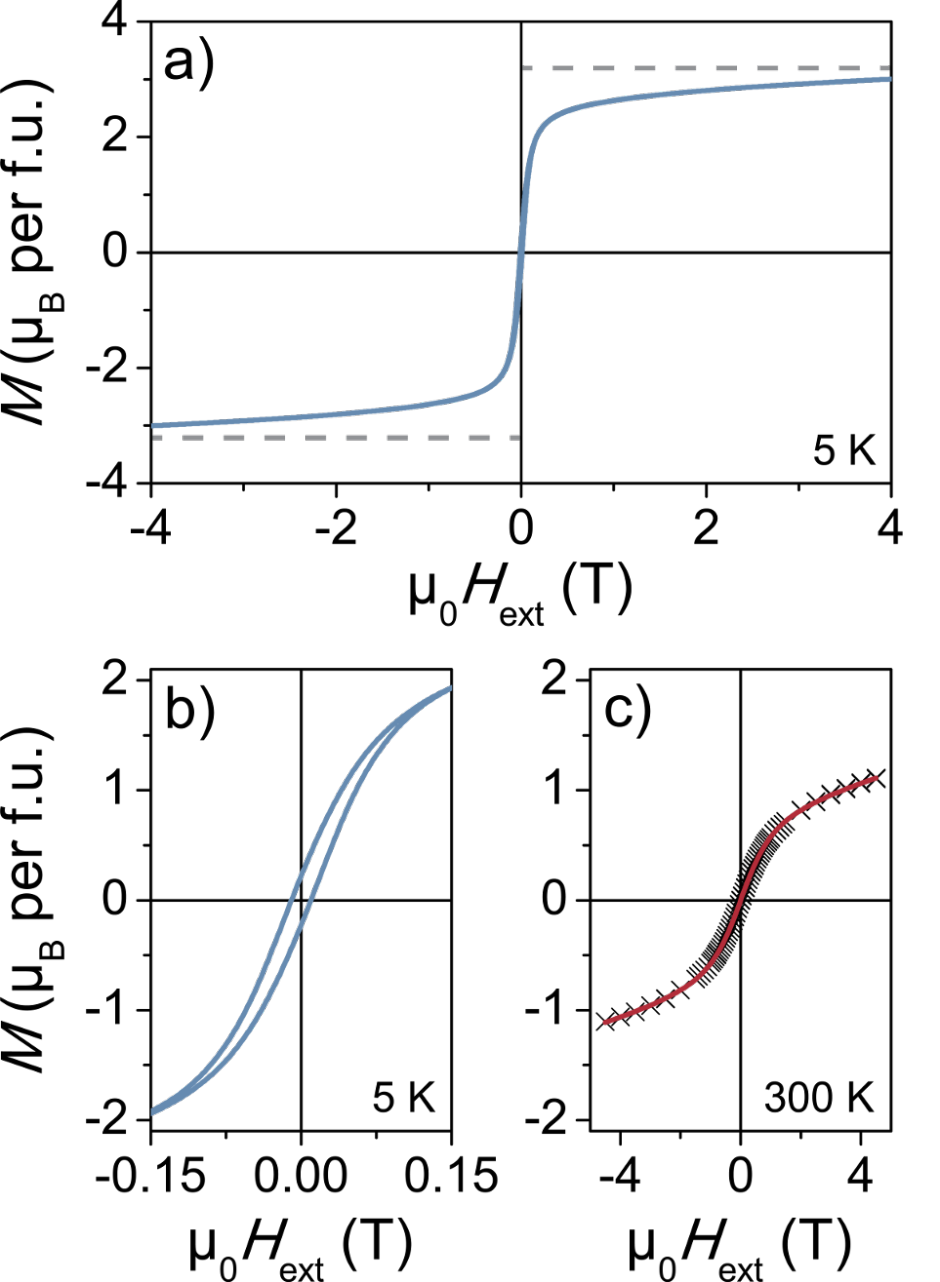
Field-dependent SQUID magnetometry of as-prepared ZFO nanoparticles at (a,b) 5 K and (c) 300 K. The dashed lines in (a) indicate the theoretical saturation magnetization. The red line in (c) is the best Langevin model fit to the data.

Figure 7a–c shows results from field-dependent SQUID magnetometry. The *M*(*H*) curve measured at 5 K (Figure 7a,b) indicates ferrimagnetic behavior with a coercive field *H*_C_ ≈ 12 mT. As evident, the magnetization is not completely saturated. Similar observations have been made for other nanocrystalline solids and are often associated with spin canting, spin freezing and so forth. Although the origin of these effects is largely unclear, they are typically attributed to magnetic frustration, surface disorder and/or finite size effects [49–50]. However, the theoretical saturation magnetization (3.2 µ_B_ per f.u.) based on the inversion parameter from XPS agrees with the experimental data. The nonlinearity of the room temperature *M*(*H*) curve in Figure 7c can be interpreted as arising from the presence of superspin glass clusters – well above their freezing temperature – in a paramagnetic environment. These data can be fitted using a simple Langevin model (*L*(*x*) with additional paramagnetic susceptibility term) of the form *M* = *M*_0_*L*(*x*) + *kH* with *L*(*x*) = coth(*x*) – 1/*x* and *x* = µ*H*/(*k*_B_*T*), where *M*_0_ is the magnetization of the superspin glass part, *k* is the paramagnetic susceptibility, µ is the magnetic moment per cluster, *k*_B_ is the Boltzmann’s constant and *T* is the temperature. The best fit was obtained with *M*_0_, *k* and µ values equal to 0.84 µ_B_/f.u., 7.8 × 10^–2^ emu/(T × f.u.) and 1090 µ_B_, respectively. Using these data and assuming a spherical cluster shape (with eight f.u. per unit cell), the cluster size was estimated to be 3.7 nm in diameter, which is in excellent agreement with the crystallite size determined by Rietveld refinement.

In a nutshell, the results from electron microscopy, XRD, XPS, ^57^Fe Mössbauer spectroscopy as well as DC and AC magnetometry are all consistent and confirm the quality of the partially inverted ZFO nanoparticles. This is also supported by the analysis of the optical properties. The Tauc plots shown in Supporting Information File 1, Figure S8 indicate an indirect band gap transition at about 650 nm (≈1.9 eV), which is in accordance with literature values and further corroborated by density functional theory calculations [51–52].

As mentioned above, spinel ferrites can, in principle, be used as negative electrode materials in rechargeable Li-ion batteries. However, they have been shown to undergo conversion at low potential and these electrochemical reactions with Li are accompanied by significant volume changes (mechanical strain), which may result in pulverization of the active material (formation of reactive surfaces) and poor cycling performance. In addition, there is usually a large hysteresis between charge and discharge, which adversely affects the energy efficiency. And this is why ZFO and other spinel ferrites are not used in commercial secondary batteries, despite high theoretical specific capacities. Nevertheless, because nanomaterials are known to better resist stresses, it was worthwhile testing the 4 nm diameter ZFO nanocrystals in Li half-cells.

**Figure 8:**
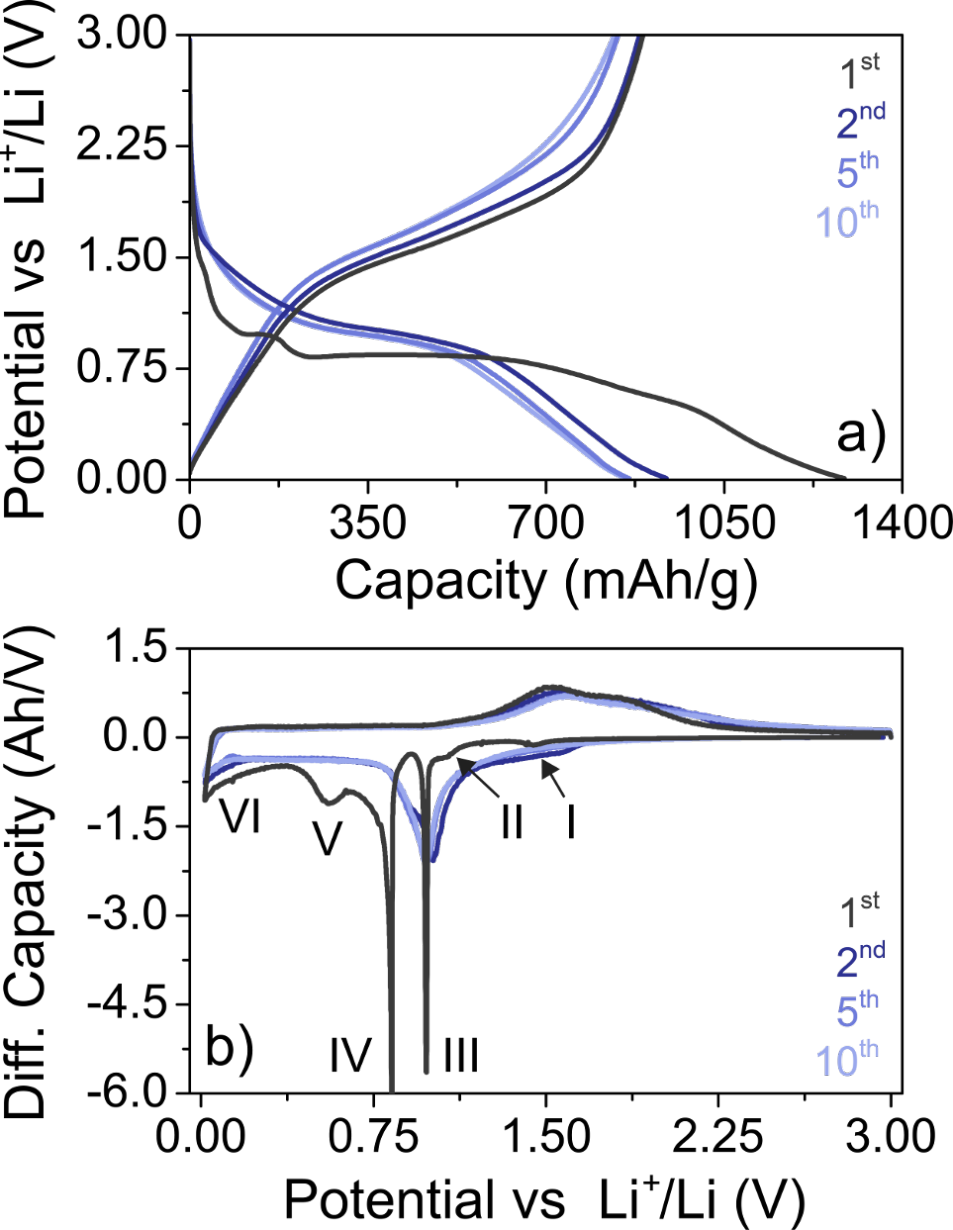
(a) Charge/discharge profiles and (b) corresponding differential capacity curves of ZFO nanoparticle electrodes in Li half-cells. The first two cycles were performed at C/20 and then the rate was increased to C/10 for the subsequent cycles. The Roman numbers in (b) indicate different electrochemical reactions in the initial cycle.

The cycling performance of electrodes having a ZFO content of 79 wt % and areal loading of 2.4 mg_ZFO_/cm^2^ in the potential range from 0.01 to 3.0 V with respect to Li^+^/Li was evaluated at different C-rates through galvanostatic charge/discharge measurements. Top view SEM images (Supporting Information File 1, Figure S9) obtained on the ZFO nanoparticle electrodes prior to cycling indicate that they are porous and there are no major structural defects and inhomogeneities, such as cracks on the micrometer level. Figure 8a shows representative charge/discharge profiles of the 1st, 2nd, 5th and 10th cycle. We note that the first two (formation) cycles were performed at C/20 before increasing the C-rate. The specific capacity in the initial cycle was always in the range of (1270 ± 20) mAh/g_ZFO_. The fact that this value exceeds the theoretical specific capacity of ZFO (*q*_th_ = 1000.5 mAh/g_ZFO_) indicates that irreversible reactions occurred upon lithiation, including decomposition of surface ligands and formation of a solid electrolyte interface (SEI) on the nanoparticles. However, this relatively large capacity loss (≈30%) was limited to the initial cycle.

The electrochemical reaction of ZFO with Li can be expressed by ZnFe_2_O_4_ + 9Li → LiZn + 2Fe + 4Li_2_O. Bresser et al. recently investigated the first cycle lithiation of ZFO nanoparticles by means of in situ XRD and correlated the result with data from charge/discharge measurements [53]. They showed that different reactions (indicated by Roman numbers in the first cycle differential capacity plot in Figure 8b) occur depending upon the potential, which is consistent with findings of others [54–56]. According to their study, the weak peak (I) at around 1.45 V can be assigned to the reversible insertion of ≈0.4 Li per f.u., while the shoulder peak (II) in the potential range from 1.2 to 1.0 V corresponds to the formation of Li_0.9_ZnFe_2_O_4_. The sharp peak (III) at 0.98 V (first plateau in Figure 8a) indicates the phase transformation from spinel to rock-salt-type Zn*_x_*Fe*_y_*O due to decomposition of Li*_x_*ZnFe_2_O_4_ (with *x* ≈ 1.5). However, we note that the underlying mechanism is not fully understood yet. The strong peak (IV) at 0.83 V can be attributed to the main conversion reaction (second plateau in Figure 8a), which results in the formation of Zn(0), Fe(0) and Li_2_O. The broad peak (V) at 0.55 V has not been observed before and likely arises due to some irreversible reactions associated with the ZFO nanoparticles. The sloping behavior of the curve (VI) below 0.4 V is characteristic of the alloying of Zn with Li [57]. In the subsequent cycles at a rate of C/10, only broad peaks centered at 0.97 V (cathodic) as well as 1.54 V and 1.85 V (anodic) were observed, which agrees with previous studies and the apparent amorphization of the material during the initial cycle [14,53,55].

**Figure 9:**
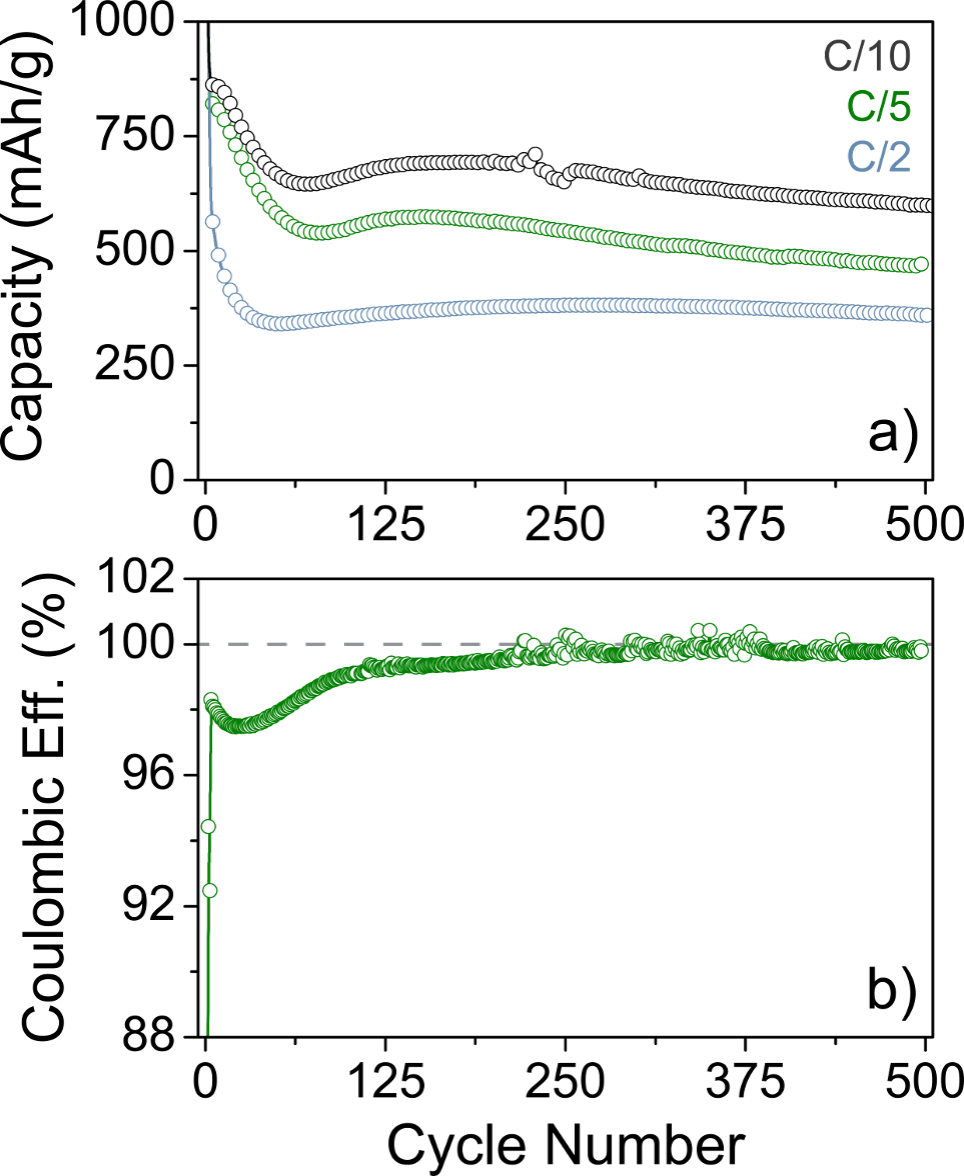
(a) Long-term cycling performance of ZFO nanoparticle electrodes in Li half-cells at C/10 (black), C/5 (green) and C/2 (blue). (b) Coulombic efficiency of the cell cycled at C/5. The dashed line in (b) indicates 100% efficiency. After about 220 cycles, signs of dendrite growth are visible in the curve.

After the first two cycles at C/20 (Figure 9a), the ZFO nanoparticle electrodes exhibited specific capacities of about 890 mAh/g_ZFO_, 870 mAh/g_ZFO_ and 770 mAh/g_ZFO_ at C/10, C/5 and C/2, respectively. Regardless of C-rate, they showed some kind of activation with a minimum in specific capacity between cycle number 50 and 80. Such behavior has been observed before for ZFO and other conversion-type anode materials [53,55,58]. The capacity degradation in the subsequent cycles – after the specific capacity had leveled off – was quite small (e.g., 0.017% per cycle at C/10). For the C/2 rate, even an increase in specific capacity by 20 mAh/g_ZFO_ was observed between the 50th and 500th cycle. After 500 cycles, the cell at C/10 rate was still capable of delivering an areal capacity of 1.5 mAh/cm^2^. These results were achieved with a non-optimized electrode structure, thereby indicating that high-quality ZFO nanocrystals may, in fact, hold promise for battery applications. We also note that dendrite growth, which was visible in some of the cells, apparently did not strongly affect the cyclability. Figure 9b shows the Coulombic efficiency of the cell cycled at C/5. As evident, the Coulombic efficiency stabilized quickly above 97% after four cycles and then increased steadily up to 99.8% by cycle number 300. This is notable in particular for conversion-type materials in Li half-cells. However, irrespective of the stable cycling performance and high specific capacities, there are still issues, such as relatively large capacity loss in the initial cycle and discontinuous capacity fading, that prevent such materials from becoming a commercial reality.

**Figure 10:**
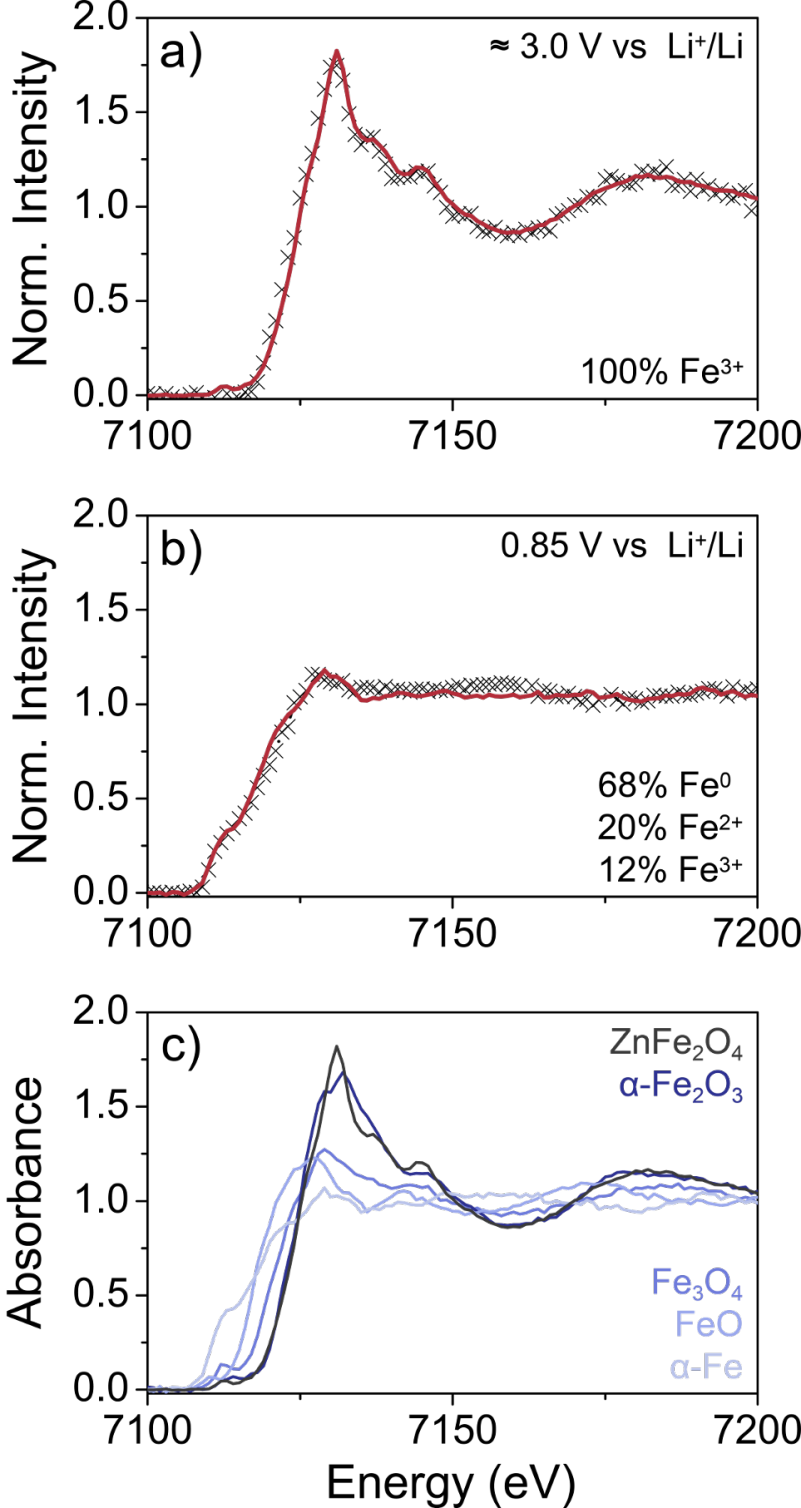
XANES spectra of ZFO nanoparticle electrodes (a) before cycling and (b) in a lithiated state and corresponding least-squares linear combination fits (red lines). (c) XANES reference spectra used for fitting.

To gain further insight into both the electrochemical reactions of ZFO with Li and the oxidation state of Fe, combined full-field transmission X-ray microscopy (TXM) and X-ray absorption near edge structure (XANES) spectroscopy was conducted on “pristine” and pre-cycled nanoparticle electrodes at the NANO beamline at the ANKA Synchrotron Radiation Facility and the preliminary data are shown in Figure 10 [25]. For these measurements, Li half-cells were disassembled inside an argon-filled glovebox and the obtained electrodes sealed using Kapton tape to maintain airtight conditions. Since XANES imaging is unaffected by the polymer binder, carbon additive, electrolyte and separator residues, the electrodes were used as is, thus ensuring minimal effects from cell disassembly. In the present work, two electrodes of the same batch but at different lithiation states were investigated. The “pristine” electrode was kept at about 3.0 V with respect to Li^+^/Li and the other was lithiated until a potential of 0.85 V was reached, which is within the main plateau. FeO (wüstite), Fe_3_O_4_ (magnetite) and α-Fe_2_O_3_ (hematite) were used as the reference materials for Fe(II) in cubic and cubic/spinel and Fe(III) in trigonal/hexagonal configuration, respectively. Furthermore, the partially inverted ZFO nanoparticles themselves and α-Fe were used as the reference materials to quantify the amount of spinel-type Fe(III) and for Fe(0), respectively. Figure 10a shows the integrated XANES spectrum obtained on the “pristine” electrode and the corresponding least-squares linear combination fit. As expected, the fit matches well with the reference material (*R* = 0.0024, χ^2^ = 0.0022), thereby indicating a ZFO content of virtually 100%. The XANES data for the pre-cycled electrode are presented in Figure 10b, where the changes in the integrated spectrum are clearly visible. The fit (*R* = 0.0021, χ^2^ = 0.0017) revealed 12% ZFO, 68% Fe(0) and 20% Fe(II) in cubic configuration. Collectively, these data are in agreement with the conversion of rock-salt-type Zn*_x_*Fe*_y_*O to Fe(0), Zn(0) and Li_2_O in this potential range. However, further measurements are needed to unambiguously identify the apparently amorphous Fe-based charge/discharge products.

## Conclusion

In summary, we have shown that zinc ferrite particles of spherical shape and uniform size around 4 nm in diameter can be prepared by facile microwave synthesis using *rac*-1-phenylethanol as a high-boiling solvent. As evidenced by electron microscopy, X-ray diffraction, X-ray photoelectron spectroscopy and ^57^Fe Mössbauer spectroscopy, the sol–gel derived material is chemically well-defined and adopts a partially inverted spinel structure. The magnetization results confirm the size monodispersity of the zinc ferrite nanocrystals with low-temperature superspin glass behavior. Furthermore, we have demonstrated that they can be used as a high-capacity conversion-type anode material, showing good long-term cycling performance in Li half-cells. On the basis of the results presented herein, we conclude that the particles are of good quality and thus hold promise for application in various fields of nanotechnology.

## Supporting Information

File 1 GC-MS, TGA-MS, Mössbauer spectra, alternating-current magnetometry and Tauc plots of as-prepared ZFO nanoparticles; SEM images of ZFO nanoparticle electrodes.
