# Supplementary material for: Microwave synthesis of high-quality and uniform 4 nm ZnFe2O4 nanocrystals for application in energy storage and nanomagnetics
Source: Beilstein J Nanotechnol. 2016 Sep 27;7:1350–60. doi: 10.3762/bjnano.7.126 (PMC5082472; doi:10.3762/bjnano.7.126)
Supplement: File 1 — GC-MS, TGA-MS, Mössbauer spectra, alternating-current magnetometry and Tauc plots of as-prepared ZFO nanoparticles; SEM images of ZFO nanoparticle electrodes. [file Beilstein_J_Nanotechnol-07-1350-s001.pdf]

Supporting Information

for

Microwave synthesis of high-quality and uniform 4 nm $ZnFe_2O_4$ nanocrystals for application in energy storage and nanomagnetics

Christian Suchomski[1,§], Ben Breitung[2,§], Ralf Witte[3], Michael Knapp[4], Sondes Bauer[5], Tilo Baumbach[5], Christian Reitz[*3] and Torsten Brezesinski[*2,3]

Address: [1]Institute of Physical Chemistry, Justus-Liebig-University Giessen, Heinrich-Buff-Ring 17, 35392 Giessen, Germany, [2]Battery and Electrochemistry Laboratory, Institute of Nanotechnology, Karlsruhe Institute of Technology, Hermann-von-Helmholtz-Platz 1, 76344 Eggenstein-Leopoldshafen, Germany, [3]Institute of Nanotechnology, Karlsruhe Institute of Technology, Hermann-von-Helmholtz-Platz 1, 76344 Eggenstein-Leopoldshafen, Germany, [4]Institute for Applied Materials, Karlsruhe Institute of Technology, Hermann-von-Helmholtz Platz 1, 76344 Eggenstein-Leopoldshafen, Germany and [5]Institute for Photon Science and Synchrotron Radiation, Karlsruhe Institute of Technology, Hermann-von-Helmholtz-Platz 1, 76344 Eggenstein-Leopoldshafen, Germany

Email: Christian Reitz - christian.reitz@kit.edu; Torsten Brezesinski - torsten.brezesinski@kit.edu

* Corresponding author

§ These authors contributed equally

GC-MS, TGA-MS, Mössbauer spectra, alternating current magnetometry and Tauc plots of as-prepared ZFO nanoparticles; SEM images of ZFO nanoparticle electrodes

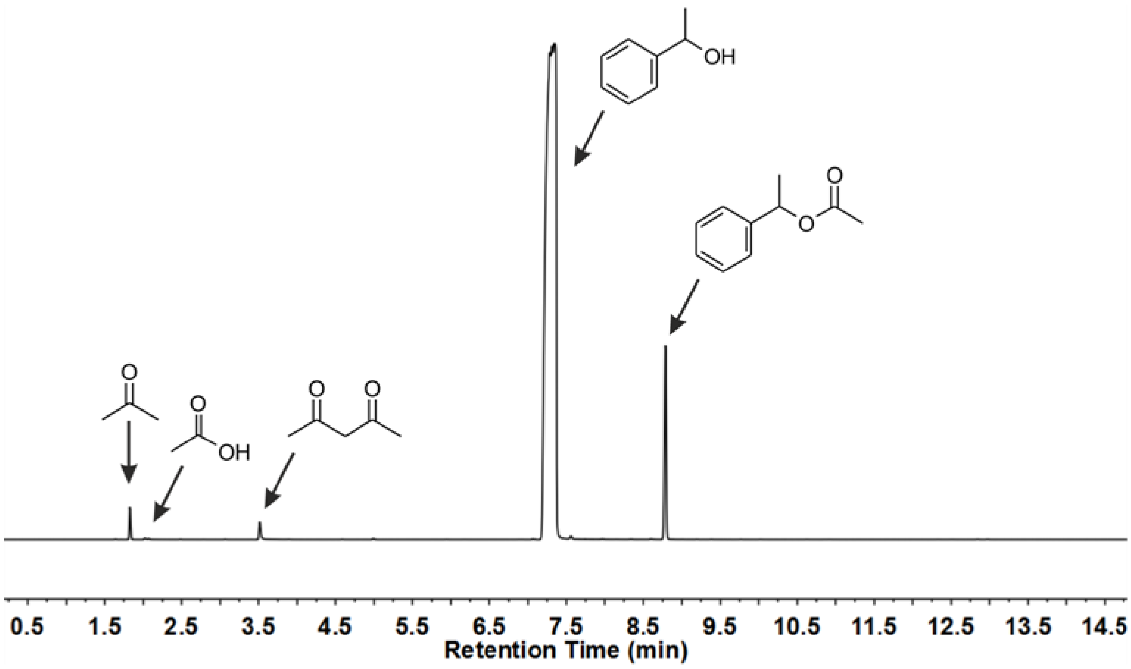

Figure S1: Gas chromatogram of the organic solution after heating at 200 °C under microwave irradiation for 25 min. Tab. S1 summarizes the GC-MS results – 1-phenylethyl acetate, acetylacetone and acetone are found to be the main organic components along with a small quantity of acetic acid.

We assume that in the first reaction step of hydroxylation zinc hydroxyacetate (Fig. S2) and iron hydroxydiacetylacetonate (Fig. S3) are generated [1–3]. In both reactions, 1-phenylethyl acetate is formed through an ester elimination, while acetone is produced by degradation of acetylacetonate *via* C−C bond cleavage and formation of an acetone-enolate complex (enolate-metal ion pair). Eventually, Fe−O−Zn bonds are generated through condensation reactions of hydroxylated iron and zinc species under release of water, acetylacetone and other compounds (Fig. S4). We note that the formation of acetophenone is not observed. This compound is found in the microwave synthesis of Fe_3O_4 nanoparticles under identical conditions. Its formation can be explained by partial oxidation of 1-phenylethanol, which is accompanied by the reduction of Fe^{3+} to Fe^{2+}, as reported elsewhere [2,4].

Table S1: Results from GC-MS analysis.

Retention time (min)	Compound	*m/z* [MP]	*m/z* [BP]	*m/z* [FP]
(1.570)	Ar, CO_2	40, 44		
1.828	acetone	58	43	42, 29, 27
2.002	acetic acid	60	43	45, 42, 15
3.520	acetylacetone	100	43	85, 72, 42
(4.994)	styrene	104	103	78, 77, 51
7.344	1-phenylethanol	122	107	79, 77, 51
7.426*	acetophenone	120	105	77, 51, 43
(7.561)	dimethyl phenyl carbinol	136	43	121, 77, 51
8.783	1-phenylethyl acetate	164	104	122, 105, 43

MP = molecule peak, BP = base peak, FP = fragment peak, *m/z* = mass/charge ratio, * not observed, () trace amount.

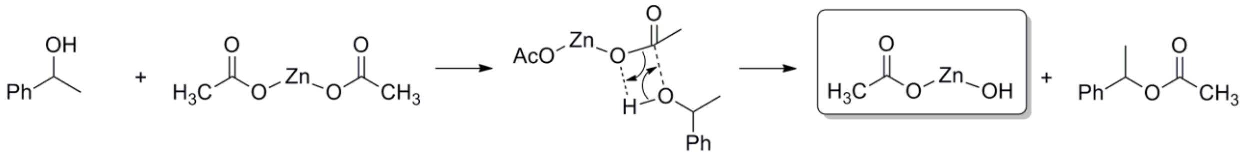

Figure S2: Formation mechanism of zinc hydroxyacetate.

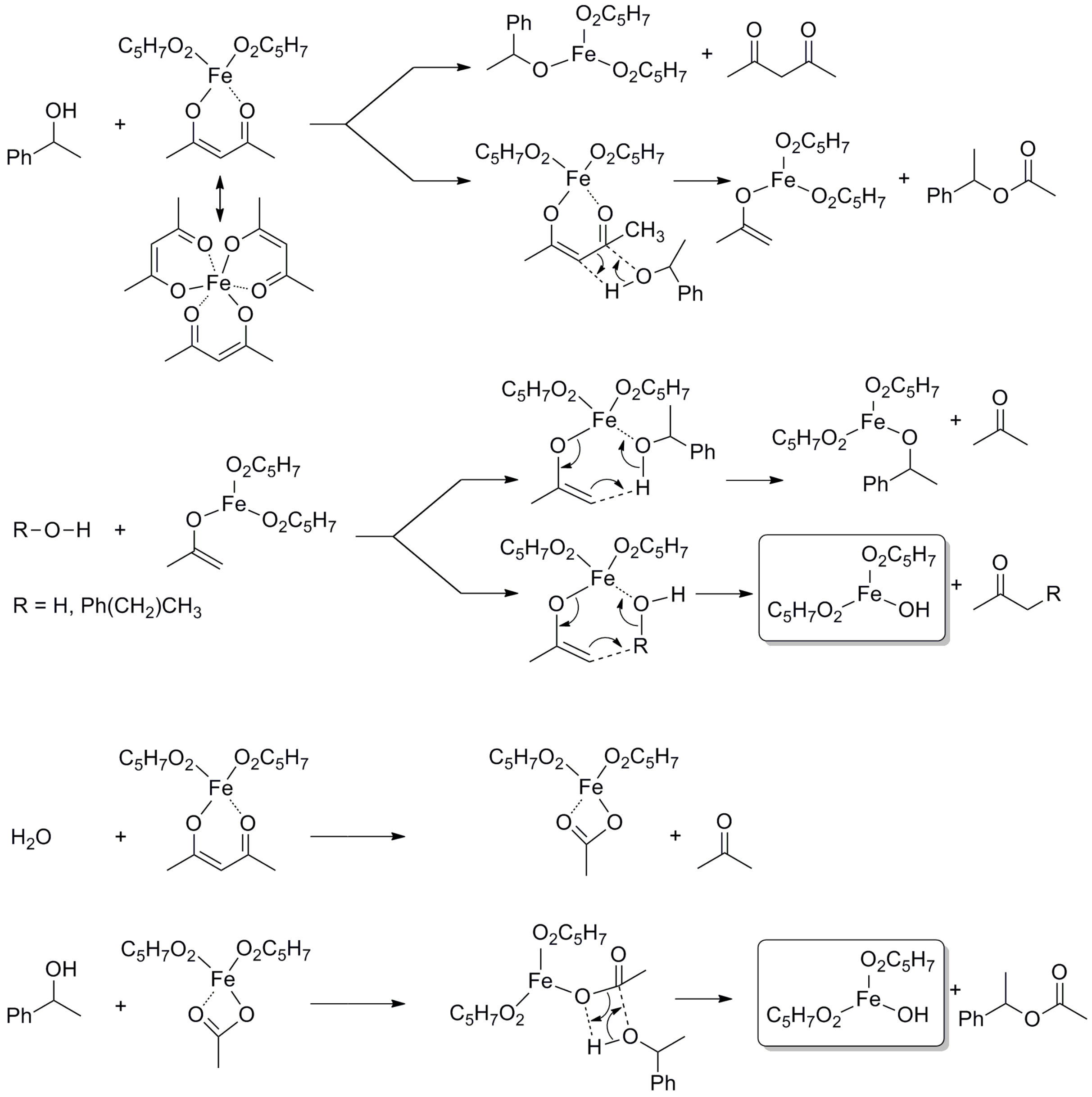

Figure S3: Formation mechanism of iron hydroxydiacetylacetonate.

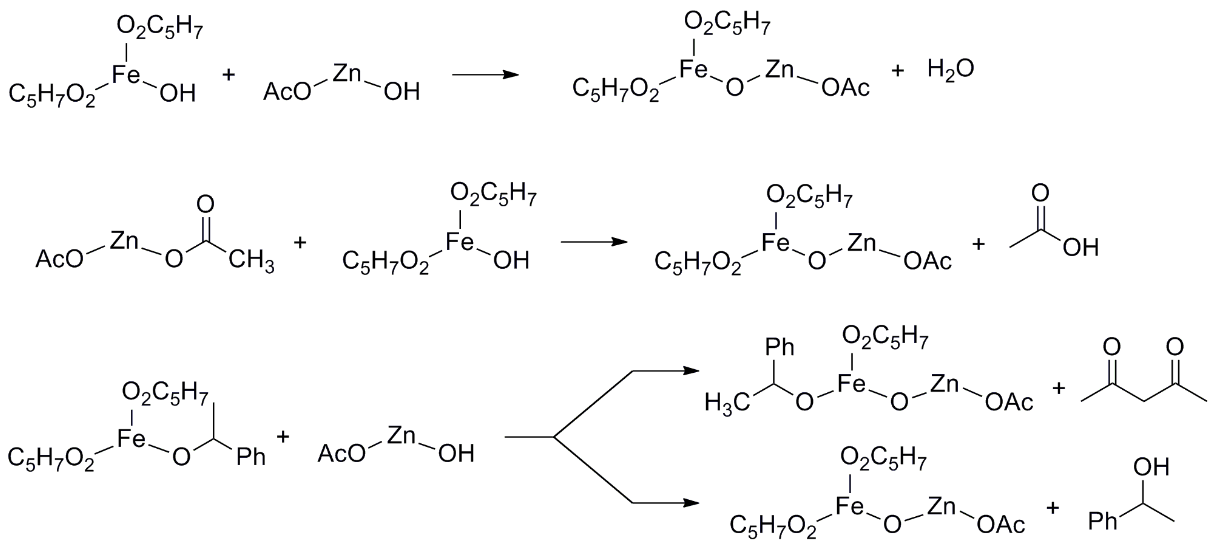

Figure S4: Condensation reactions of in situ generated iron and zinc species.

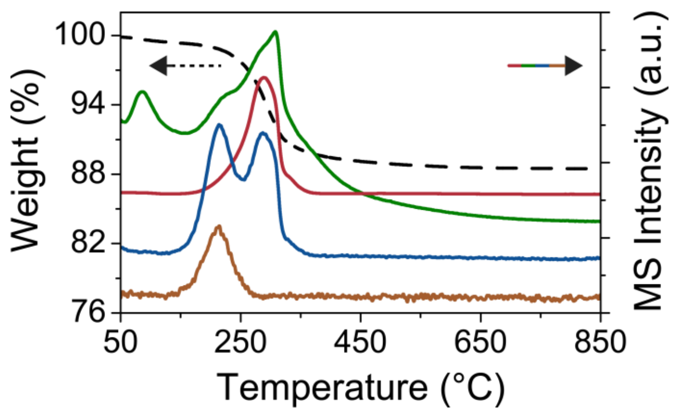

Figure S5: TGA-MS of room temperature vaccum-dried ZFO nanoparticles in synthetic air at 5 °C/min. The dashed black line is the TG curve. The MS analysis shows H_2O (m/z = 18) in green, CO_2 (m/z = 44, $I \times 0.25$) in red, acetyl (m/z = 43, $I \times 50$) in blue and acetone (m/z = 58, $I \times 200$) in brown. The data indicate a total mass loss of 13% by 850 °C. The combustion of organic species, namely acetate and acetylacetonate ligands, occurs in the temperature range between 150 °C and 400 °C.

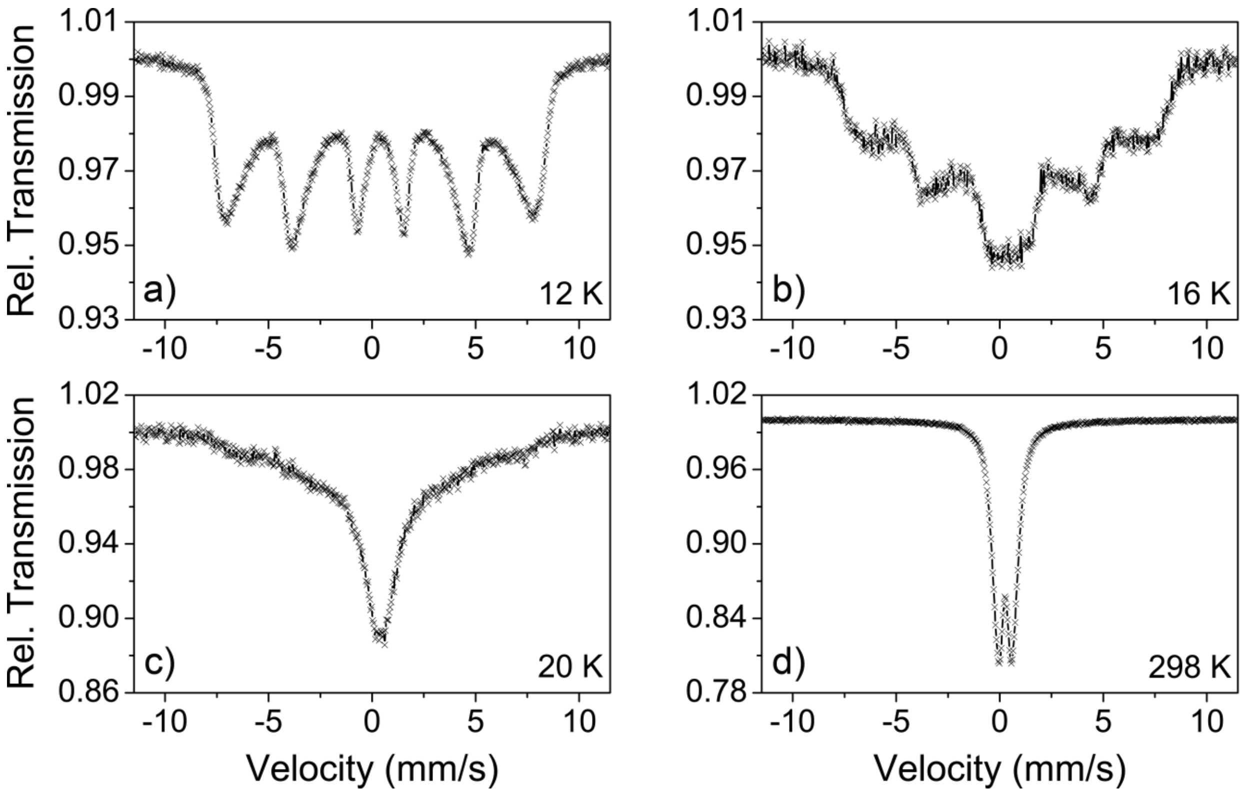

Figure S6: Mössbauer spectra of as-prepared ZFO nanoparticles measured at 12 K (a), 16 K (b), 20 K (c) and 298 K (d). The magnetic coupling begins to collapse at about 12 K (strong line broadening), but is still present to some extent up to 20 K. Overall, the data agree with the results from temperature-dependent SQUID magnetometry. The doublet line shape observed at 298 K is indicative of the loss of magnetic ordering.

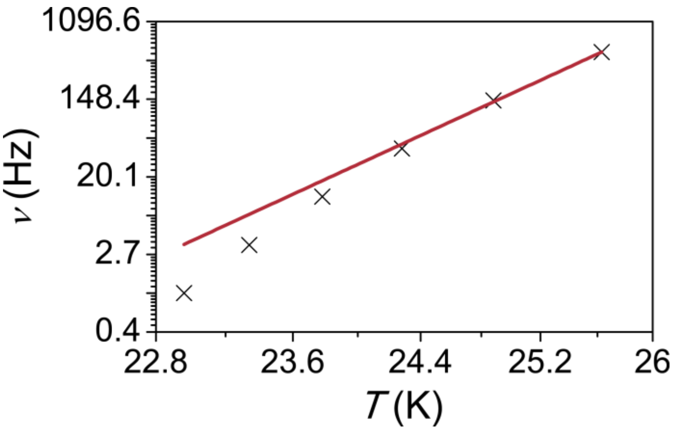

Figure S7: Alternating current SQUID magnetometry (with $\mu_0 H_{AC}$ = 0.35 mT) of as-prepared ZFO nanoparticles. As seen, the Néel–Brown relation ($\nu = \nu_0 \times \exp[KV/k_B T]$) for ideal non-interacting superparamagnetic particles cannot be used to describe the data properly.

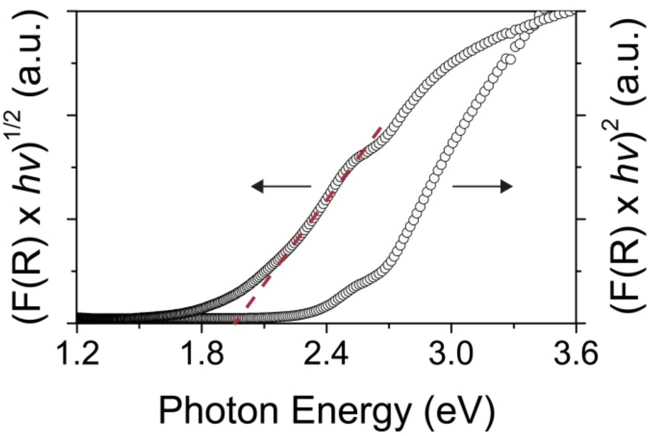

Figure S8: Direct and indirect optical transitions in as-prepared ZFO nanoparticles. The Tauc plots provide information on the band gap energy, E_g, and the characteristic shape of the curve of $(F(R) \times hv)^{1/2}$ vs. photon energy reveals an indirect transition with $E_g = (1.90 \pm 0.05)$ eV at room temperature (see intercept of the dashed red line with the *x*-axis).

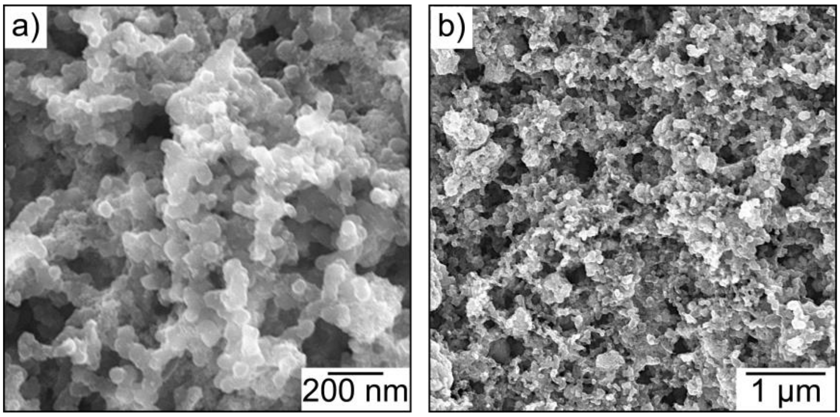

Figure S9: Top view SEM images at different magnifications of ZFO nanoparticle electrodes prior to cycling.

References

1. Joo, J.; Kwon, S. G.; Yu, J. H.; Hyeon, T. *Adv. Mater.* **2005**, *17*, 1873–1877. doi:10.1002/adma.200402109

2. Niederberger, M.; Garnweitner, G. *Chem. Eur. J.* **2006**, *12*, 7282–7302. doi:10.1002/chem.200600313

3. Rudolph, G.; Henry, M. C. *Inorg. Chem.* **1964**, *3*, 1317–1318. doi:10.1021/ic50019a026

4. Staniuk, M.; Zindel, D.; van Beek, W.; Hirsch, O.; Kränzlin, N.; Niederberger, M.; Koziej, D. *CrystEngComm* **2015**, *17*, 6962–6971. doi:10.1039/C5CE00454C
